# Protection of acute myeloid leukaemia cells from apoptosis induced by front-line chemotherapeutics is mediated by haem oxygenase-1

**DOI:** 10.18632/oncotarget.321

**Published:** 2011-09-09

**Authors:** Sally-Anne Heasman, Lyubov Zaitseva, Kristian M. Bowles, Stuart A. Rushworth, David J. MacEwan

**Affiliations:** ^1^School of Pharmacy, University of East Anglia, Norwich Research Park, Norwich, NR4 7TJ, UK; ^2^Department of Haematology, Norfolk and Norwich University Hospital NHS Trust, Colney Lane, Norwich, NR4 7UY UK

**Keywords:** drug-resistance, antioxidant, transcription factor, apoptosis, chemotherapy, MicroRNA

## Abstract

Haem oxygenase-1 (HO-1) is increasingly regarded as a pro-tumoral target in the treatment of human cancers. Currently, little is known about HO-1 and its role in human acute myeloid leukaemia (AML) to regulate apoptosis in response to chemotherapy. Recently, we showed that HO-1 protects AML samples from tumour necrosis factor-α (TNF)-induced apoptosis - it being regulated by transcription factors Nrf2, NF-κB and AP-1. This study aims to analyse the role of HO-1 in regulating apoptosis in AML cells in response to two front-line chemotherapeutic agents used for AML, cytarabine and daunorubicin. Here we show that HO-1 expression in AML samples was increased in response to both cytarabine and daunorubicin treatment, and micro RNA (miRNA) silenced HO-1 expression in combination with either daunorubicin or cytarabine induced a greater apoptotic responses in AML cells. Moreover, we showed that both daunorubicin and cytarabine induced reactive oxygen species (ROS) accumulation to induce apoptosis in AML. However, ROS-dependent induction of HO-1 was limiting the apoptotic response that is seen in AML towards cytarabine and daunorubicin treatment. These findings suggest concurrent inhibition of HO-1 expression in conjunction with chemotherapeutic treatment would improve the number of cases who reach complete remission.

## INTRODUCTION

Acute myeloid leukaemia (AML) is a malignancy of haemopoietic progenitor cells [1] and accounts for approximately 1% of all cancer deaths. At present the standard induction treatment for younger fitter patients consists of the antimetabolite cytarabine plus the anthracycline antibiotic daunorubicin [2, 3]. Depending on clinicopathological characteristics, patients who go into remission would commonly receive consolidation therapy with either high dose cytarabine or allogeneic stem cell transplant. Despite these intensive treatment strategies, significant numbers of patients relapse and only approximately 50% of younger fitter patients can be cured. The treatment outcomes are dependent on a variety of clinical and biological factors including cytogenetics, age and drug-resistance [2, 4-7].

A number of mechanisms have been suggested to contribute to drug-resistance in AML. These include, the targeted cells fail to undergo apoptosis in response to the chemotherapy agent, drugs failing to reach their intracellular targets or the removal by the ABC membrane transporter protein, P-glycoprotein (Pgp). Pgp is a efflux transporter, present within the cell's plasma membrane, and its expression in AML has been reported to be relatively low, however an increase in its expression after drug treatment and also at the point of relapse, have been reported [8]. Moreover, Galmarini et al have shown that high levels of 5′-nucleotidase, which is involved in DNA repair and membrane transport, is related to the poor prognosis of AML patients [9]. These studies suggest that AML cells evolve to regulate pathways that provide protection against toxic chemotherapeutic agents.

Recently, we reported that haem oxygenase-1 (HO-1) has an important function in protecting human AML cells from TNF-induced apoptosis [10]. To date, three isoforms of haem oxygenase have been identified, HO-1, HO-2 and HO-3 [11]. HO-1, which is the most intriguing in terms of providing protection against cellular stresses, regulates cellular haem levels, and converts intracellular haem into carbon monoxide, free iron and biliverdin [11]. Biliverdin is further reduced into the potent antioxidant bilirubin [12, 13] by biliverdin reductase [14]. This metabolite possesses cytoprotective properties including anti-inflammatory, anti-oxidative and anti-apoptosis [15, 16]. HO-2 is constitutively expressed and HO-3 is not catalytically active and thought to be involved in oxygen-sensing. HO-1 belongs to the heat shock protein family (Hsp-32), thus its expression is triggered by a variety of stress-inducing stimuli including, UV irradiation, hyperthermia, inflammatory cytokines, bacterial endotoxins and heavy metals [17-21]. The regulation of HO-1 is under the control of signalling components [22, 23] and many transcription factors including nuclear factor-κB (NF-κB), NF-E2-related factor 2 (Nrf2) and activator protein-1 (AP-1)[24, 25].

Furthermore we have recently we showed that HO-1 is in fact down-regulated in AML by their constitutively active NF-κB activity present, and that inhibiting NF-κB brings HO-1 levels back to more normal levels, providing further secondary protection for AML cells against NF-κB inhibition. Here were undertaken to investigate the role of HO-1 in regulating cytoprotective responses to two common front-line chemotherapy agents, cytarabine and daunorubicin, which are currently widely used in treating patients presenting with AML.

## RESULTS

### AML resistance to cytarabine and daunorubicin

**Figure 1:**
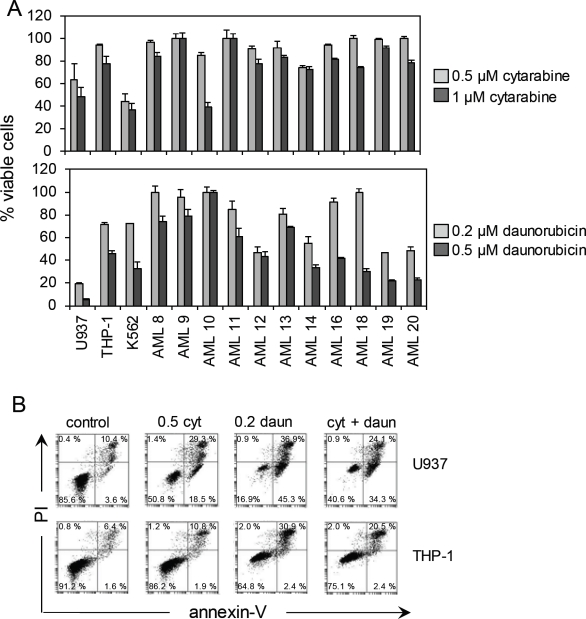
AML cell resistance to cytarabine and daunorubicin (A) AML cells were treated with two doses of cytarabine (0.5 μM and 1 μM) and two doses of daunorubicin (0.2 μM and 0.5 μM) for 24 hours. Viable cell numbers were then devised using PI and annexin V staining followed by flow cytometry. Values indicate mean ± SD from three separate independent experiments. (B) U937 and THP-1 cells were treated with either cytarabine 0.5 μM, daunorubicin 0.2 μM or a combination of cytarabine 0.5 μM and daunorubicin 0.2 μM for 24 hours. Samples were stained with annexin V and PI. Flow cytometry analysis was used to determine the percentage of viable cells present within the sample population.

To understand the mechanisms of chemoresistance in AML cells to cytarabine and daunorubicin, we have examined the levels of apoptosis of primary AML samples and AML cell lines in response to varying concentrations of these drugs. The drug concentrations selected were based on previous studies [26, 27]. One μM of cytarabine is a clinically achievable concentration obtained in situations where a standard dose of cytarabine is administered [28]. Table 1 shows the relevant clinical data for the AML patient samples tested in these studies. Figure 1A shows apoptosis for both AML samples and AML cell lines in response to both cytarabine (0.5 μM and 1 μM) and daunorubicin (0.2 μM and 0.5 μM) in concentration-dependent manners. Figure 1B shows that cytarabine (0.5 μM), daunorubicin (0.2 μM) or a combination of both cytarabine and daunorubicin induce cell death by apoptosis as measured by annexin-V and PI staining. What is clear from these findings is that there is a range of sensitivities to the chemotherapeutics and that there are significant levels of living cells that remain resistant to these cytotoxic agents, even in combination.

**Table 1:** Characteristics of study patient samples AML disease characteristics including WHO diagnosis and cytogenetics. Percent blast denotes % of AML blasts after purification using density gradient (* denotes % of blasts and promyelocytes). Previous treatments are as outlined [29]. Abbreviations used: AML, Acute myeloid leukaemia; WHO, world health organisation.

Number	Age	Gender	WHO diagnosis	Cytogenetics	% Blasts	Previous treatment
**AML8**	40	male	Acute promyelocytic leukaemia with t(15;17)(q22:q12) PML-RARA	t(15;17)	95*	1999 DAT,DAT MACE,MiDAC [29]
**AML9**	49	male	AML with maturation	normal	80	nil
**AML10**	84	male	Acute monoblastic and monocytic leukaemia	not available		nil
**AML11**	46	female	AML with maturation	+4,+8, t(9;22)	70	nil
**AML12**	78	male	AML with myelodysplasia related changes	not available	85	nil
**AML13**	27	male	AML with t(8;21)(q22;q22) RUNX1-RUNX1T1	t(8;21)		nil
**AML14**	28	female	Acute myelomonocytic leukaemia	normal		nil
**AML16**	66	female	therapy related myeloid neoplasm	complex	85	1999 DAT,DAT MACE,MiDAC [29]
**AML18**	77	male	AML with myelodysplasia related changes	complex	95	nil
**AML19**	31	female	AML with minimal differentiation	normal		nil
**AML20**	40	male	Acute monoblastic and monocytic leukaemia	constitutional XYY only	90%	nil

### Cytarabine and daunorubicin induce HO-1 expression in AML cells

**Figure 2:**
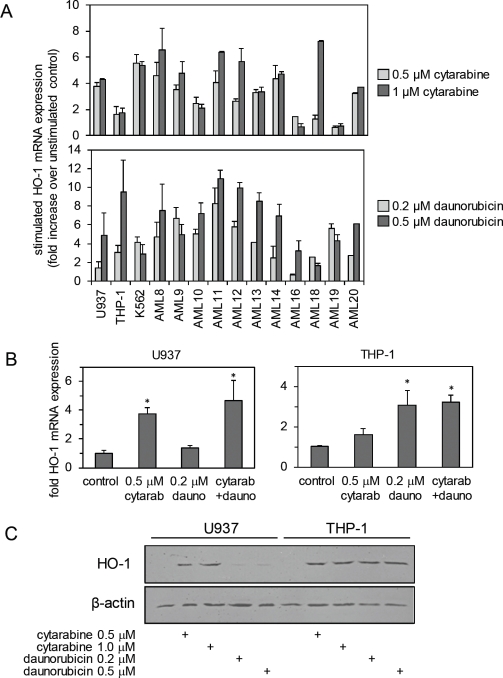
Cytarabine and daunorubicin induce HO-1 expression in AML cells (A) AML cells were treated with cytarabine (0.5 μM or 1 μM) and daunorubicin (0.2 μM or 0.5 μM) for 24 hours. RNA was extracted from each sample and HO-1 mRNA was measured using real-time PCR. (B) U937 and THP-1 cells were treated with either cytarabine 0.5 μM, daunorubicin 0.2 μM or a combination of both cytarabine 0.5 μM and daunorubicin 0.2 μM for 24 hours. RNA was extracted from each sample and HO-1 expression was measured using real-time PCR. (C) U937 and THP-1 cells were treated with either cytarabine (0.5 μM or 1 μM) or daunorubicin (0.2 μM or 0.5 μM) for 24 hours. Whole cell protein extracts were collected and separated via SDS-PAGE and examined using Western immunoblotting analysis. HO-1 expression levels were examined. Membranes were reprobed with β-actin to ensure equal loading across the samples.

Since we have previously shown that HO-1 protects AML cells from apoptotic stimuli, we wanted to determine if either cytarabine or daunorubicin could induce the expression of this cytoprotective gene in AML cells. Figure 2A shows that both cytarabine and daunorubicin induce HO-1 mRNA expression by 24 h of drug exposure in both AML cell lines and AML patient samples. Figure 2B also reiterates the above point in AML cell lines and shows the effect on HO-1 mRNA expression of 24 h drug exposure to cytarabine and daunorubicin alone or in combination. Interestingly, HO-1 mRNA expression is increased further when the cells are exposed to both chemotherapeutic agents together. Figure 2C shows a Western blot indicating that HO-1 protein was also induced by both cytarabine and daunorubicin after 24 h drug exposure. Thus these front-line chemotherapeutic agents are able to readily induce HO-1 in AML cells. Furthermore, at the concentrations used, daunorubicin is somewhat better at killing off U937 cells than is cytarabine (Figure 1), with daunorubicin only weakly inducing HO-1 in U937 cells compared to cytarabine's induction (Figure 2C). This correlates perfectly with a role for HO-1 induction in drug-resistance in U937 cells. Likewise, it is notable that both agents can equally induce strong HO-1 expression in THP-1 cells, with a similar inability towards efficient cytotoxicity in those cells.

### Silencing HO-1 increases AML cell apoptosis to cytarabine and daunorubicin

**Figure 3:**
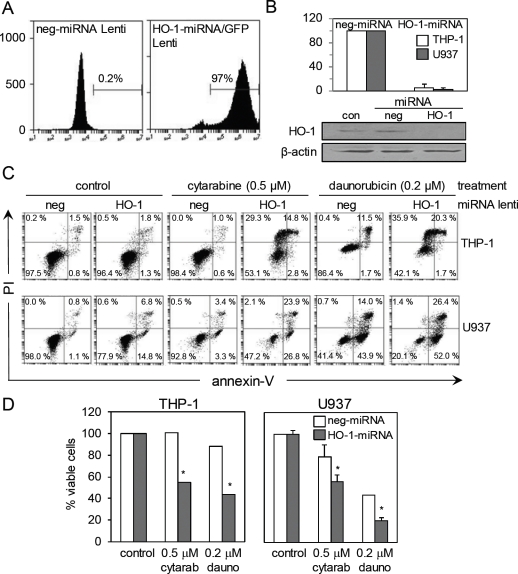
Silencing HO-1 increases AML cell apoptosis to cytarabine and daunorubicin (A) Flow cytometry was used to measure the transfection efficiency of the lentivirus based miRNA HO-1/GFP construct (B) U937 and THP-1 cells were infected with a lentiviral based miRNA HO-1/GFP knockdown construct, whilst the control was infected with lentiviral based miRNA negative construct. RNA was extracted from each sample and HO-1 mRNA expression was measured using real-time PCR. (C) Whole cell protein extracts were collected and separated via SDS-PAGE and examined using Western immunoblotting analysis to investigate HO-1 expression. (D) Both control U937 and THP-1 cells and HO-1 silenced U937 and THP-1 cells were treated with cytarabine 0.5 μM and daunorubicin 0.2 μM for 24 hours. Cells were stained with annexin-V and PI, the percentage of viable cells was measured via flow cytometry.

We developed a lentivirus-based miRNA delivery system on a GFP backbone to target HO-1 mRNA knockdown and silence HO-1 expression in AML cells. Figure 3A shows that we can achieve > 95% transfection efficiency as measured by flow cytometry. Figure 3B shows that HO-1 mRNA expression in both THP-1 and U937 control and HO-1-silenced cells, with greater than 95% knock-down of endogenous HO-1 mRNA levels observed. Figure 3B also indicates HO-1 protein expression in THP-1 control and HO-1-silenced cells, with almost no detectable levels of HO-1 observed specifically with the HO-1 miRNA lentivirus. Next we wanted to determine if either cytarabine or daunorubicin induced HO-1 expression could protect AML cells from apoptosis. To do this we used lentiviral-based miRNA HO-1 knockdown cells (compared to control negative miRNA lentiviral knockdown cells) treated with either cytarabine or daunorubicin for 24 h. Figure 3Cand 3D show the apoptotic responses of control- and HO-1-silenced THP-1 and U937 cells after treatment with either cytarabine or daunorubicin. The HO-1-silenced THP-1 and U937 cells were more susceptible to apoptosis compared to the non-silenced cells. These findings indicate that HO-1 is playing a part in cellular protection mechanisms observed in apoptosis-resistance towards cytarabine or daunorubicin.

### ROS modulates the apoptotic potential of cytarabine and daunorubicin in AML cells

**Figure 4:**
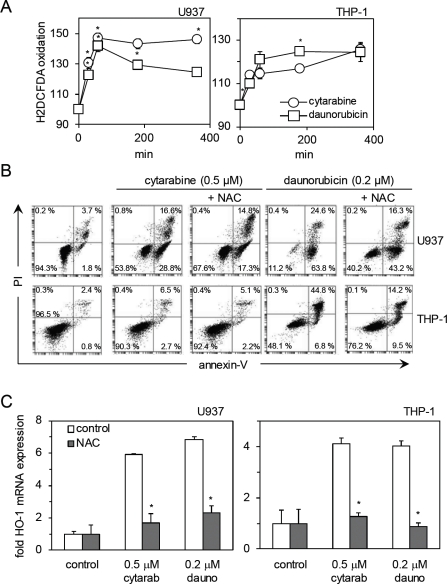
Reactive oxygen species (ROS) modulate the apoptotic potential of cytarabine and daunorubicin in AML cells (A) U937 and THP-1 cells were treated with cytarabine 0.5 μM and daunorubicin 0.2 μM for up to 6 hours and then washed with PBS and incubated for 15 minutes with 10 μM of H_2_DCFDA. ROS production in response to drug exposure was examined using flow cytometry. (B) U937 and THP-1 cells were treated with cytarabine 0.5 μM and daunorubicin 0.2 μM for 24 hours with or without pre treatment with NAC (10 μM for daunorubicin treated cells and 5 μM for cytarabine treated cells) for 30 minutes. Cells were stained with annexin-V and PI, their response to NAC was examined using flow cytometry. (C) U937 and THP-1 cells were treated with either cytarabine 0.5 μM and NAC 5 μM or daunorubicin 0.2 μM and NAC 10μM for 24 hours. RNA was extracted and mRNA HO-1 expression was measured using real- time PCR.

We have previously shown in AML cells that H_2_DCFDA oxidation occurs in response to the proteasome inhibitor bortezomib and also in response to NF-κB inhibitor BAY 11-7082 [29, 30]. We have also shown that ROS induced by these drugs are responsible for upregulating HO-1 expression. Here we wanted to determine if cytarabine and daunorubicin increased HO-1 expression through a ROS-dependent mechanism. Figure 4A shows H_2_DCFDA oxidation in U937 and THP-1 cells in response to cytarabine and daunorubicin treatment over a 6 h time period. N-acetylcysteine (NAC) is an antioxidant scavenger which quenches ROS activity present within the cellular environment. NAC was used to determine if removing ROS would block cytarabine- and daunorubicin-induced HO-1 expression. Finally, we wanted to determine whether NAC could block apoptosis induced by cytarabine and daunorubicin. Figure 4B show that NAC effectively inhibits apoptosis in cytarabine- and daunorubicin-treated THP-1 and U937 cells, thus demonstrating that ROS are responsible for cytarabine- or daunorubicin-induced cell death of AML cells. Figure 4C shows a decrease in mRNA HO-1 expression in the presence of NAC compared to that of the control, further implicating ROS in the chemotherapeutic-induction of HO-1 and its consequent cytoprotection observed in AML.

## DISCUSSION

In this study we have investigated the role of HO-1 in protecting AML cells from apoptosis in response to cytarabine and daunorubicin. We have previously shown that HO-1 can protect AML cells from apoptosis in response to the proteosome inhibitor, bortezomib and the NF-κB inhibitor, BAY11-7082 [29, 30]. In the present work, we show that expression of HO-1 is induced in response to cytarabine, daunorubicin and a combination of both agents. Interestingly HO-1 mRNA expression is increased further when the cells are treated with a combination of the drugs which one would expect because of the presence of both agents acting via differing modes of action to induce apoptosis. Referring to Figure 1B, oddly the percentage of viable cells present within the sample population is actually between that of separate cytarabine and daunorubicin exposure. Furthermore silencing HO-1 expression in combination with either cytarabine or daunorubicin induces a greater apoptotic response in AML cells. Finally we show that both cytarabine and daunorubicin cause AML cells to generate ROS production and this in turn induces HO-1 expression, which subsequently reinforces any cellular resistance towards these drugs. A prior report has already shown ROS production in the presence of cytarabine in U937 cells [26]. These results provide novel insight into the role of HO-1 in protecting human AML cells from cytarabine and daunorubicin.

In a broad range of morphologic and cytogenetic subtypes of primary human AML cells and human AML cell lines, both cytarabine and daunorubicin consistently induce HO-1 expression by 24 h. We also examined the effects of daunorubicin and cytarabine on HO-1 expression at earlier time points finding no increase in HO-1 mRNA levels (not shown). This time lag between addition of drug and HO-1 induction in AML cells is interesting. We hypothesise that this is likely to be due to the complex nature of the HO-1 promoter and its regulation. Nuhn et al have reported that HO-1 expression is usually increased in human cancer cells and is also increased further by the addition of cytotoxic agents [31]. Subsequent silencing of HO-1 in these AML cell lines significantly decreases the percentage of viable AML cells present within the population treated with fixed doses of either cytarabine or daunorubicin. Therefore we suggest inhibiting HO-1 could potentially enhance AML cell response to these chemotherapeutic agents in vivo. Fang et al have determined the presence of a HO-1 inhibitor in vivo can enhance tumour responsiveness to chemotherapeutic agents [32].

Protection from apoptosis conferred to AML cells by HO-1 in the current study appears to be medicated through regulation of ROS produced in response to cytarabine and daunorubicin exposure. With regards to HO-1 and its ability to protect cells from undergoing apoptosis, there are a number of different proposed mechanisms. The first is by decreasing the levels of the pro-oxidant, haem [32, 33]; the second, by increasing the concentration of bilirubin, an antioxidant [32]; and the final mechanism is by increasing the levels of carbon monoxide, curiously generally thought to be an anti-apoptotic molecule [13, 34, 35]. Together, these mechanisms act either alone or in a combined way to protect cancer cells from apoptosis. What is intriguing about AML cells is that they seem to have evolved to manipulate this pathway to provide it with a growth advantage over normal non-cancerous control cells. AML (or any cancerous cell) probably doesn't set out to be drug-resistant, but there is a varying population of cancer stem cells that are relatively able to cope with any cytotoxic insult they receive from cytarabine or daunorubicin. We show here that these resistance mechanisms heavily involve HO-1. A patient's HO-1 response to chemotherapeutics will go a long way in determining their clinical outcome.

In our previous study we showed that basal levels of HO-1 are low compared to normal cells and that NF-κB activation reduced basal HO-1 expression. Furthermore, Nrf-2 activation could override this regulation [29]. In fact any number of transcription factors can regulate HO-1 expression [24, 25]. In this study we concentrated on the role of HO-1 expression in regulating chemotherapy-induced apoptosis but cannot preclude the involvement of other transcriptional elements.

In summary we report that silencing HO-1 significantly increases in vitro sensitivity of AML cells to cytarabine and daunorubicin, two front-line chemotherapy agents currently widely used to treat this type of leukaemia. Previously we have shown that HO-1 plays an important role in protecting AML cells from apoptotic stimuli and here we propose that this is likely to be clinically relevant and thus HO-1 warrants further investigation as a target in future therapeutic strategies.

## MATERIALS AND METHODS

### Materials

The AML-derived cell lines THP-1 and U937, were obtained from the European Collection of Cell Cultures, where cell identity is confirmed by cytogenetic analuysis. Cells were used in the laboratory for a maximum of 6 months post-revival from liquid nitrogen storage, to ensure integrity and retention of characteristics. Anti-human HO-1 antibody was purchased from R & D Systems (Abingdon, UK). All other antibodies were obtained from Abcam (Cambridge, UK). All primers were purchased from Invitrogen (Paisley, UK). Micro RNA (miRNA) vector was cloned on site. Annexin-V and propidium iodide (PI) were purchased from Abcam (Cambridge, UK). Cytarabine (dissolved in water, stored at −20 °C as a 4.1 mM stock) and daunorubicin (dissolved in water, stored at −20 °C as a 0.95 mM stock) were both purchased from Sigma-Aldrich (St Louis, MO). All other reagents were purchased from Sigma- Aldrich unless stated.

### Cell Culture

Primary AML cells were obtained under local ethical approval (LREC ref 07/H0310/146). For primary cell isolation, heparinised blood was collected from volunteers and isolated by Histopaque (Sigma-Aldrich, St Louis, MO) density gradient centrifugation. U937 were cultured in a humidified atmosphere at 37°C with 5% CO_2_ in RPMI-1640 from Invitrogen (Paisley, UK) supplemented with 10% foetal bovine serum (FBS) (Biosera, UK) and 1% L-glutamine incubated. 293FT cells were obtained from Invitrogen and were grown in DMEM high glucose (Invitrogen) supplemented with 10% FBS, 6 mM L-glutamine, 0.1 mM non-essential amino acids and 1 mM sodium pyruvate.

### RNA extraction and real-time PCR

Total RNA was extracted from 1 × 10^6^ cells using RNA lysis solution and a nucleic acid Prepstation both purchased from Applied Biosystems (Warrington, UK) following the manufactures instructions. Applied Biosystems RNA PCR core kit was used for reverse transcription. SYBR green technology (Roche) was used on cDNA produced via the reverse transcription of purified RNA. After pre-amplification (95 °C for 2 min) the reactions were amplified for 45 cycles (95°C for 15 s, then 60°C for 10 s, followed by 72°C for 10 s) on a Lightcycler 480 (Roche) as described [29]. HO-1 mRNA expression was standardised against GAPDH expression using the standard curve analysis method. Primer sequences were: GAPDH forward 5′-ACCAGCCTCAAGATCATCAGC-3′; GAPDH reverse 5′-TGCTAAGCAGTTGGTGGTGC-3′; HO-1 forward 5′-ATGGCCTC CCTGTACCACATC-3′ and HO-1 reverse 5′-TGTTGCGCTCAATCTCCTCCT-3′.

### Western immunoblotting

Total protein was extracted from 1 × 10^6^ cells using radioimmunoprecipitation assay (RIPA) buffer as described [29]. Protein was transferred onto nitrocellulose membrane, probed according to the antibody manufacturer's guidelines and then examined using enhanced chemiluminescence (ECL) detection in a Li-Cor Odyssey IR imaging system.

### Flow Cytometry

Flow cytometry for measuring apoptosis was performed on an Accuri C6 flow cytometer (Accurri). Samples were collected, centrifuged at 500 × g, 5 min then resupended and stained with Annexin-V and PI dye, followed by detection. N-acetylcysteine (NAC) antioxidant ROS-scavanger was used to assess the presence of ROS, whereby cells were treated with NAC with relevant chemotherapeutic agent for 24 h, prior to sample flow cytometry analysis.

### Dichlorofluorescein Assay

A dichlorofluorescein (DCF) assay was used to determine cellular ROS generation [21]. Cells were washed with PBS and then incubated with 10 μM of 2′,7-dichlorodihydrofluorescein diacetate (H_2_DCFDA) for 15 min at 37 °C and 5% CO_2_. Flow cytometry was used to measure the fluorescence intensity of H_2_DCFDA. The mean channel fluorescence of a treated sample is divided by that of the control and then multiplied by 100, thus obtaining the relative percentage change. The relative fluorescence intensity of H_2_DCFDA detected represents the steady state of ROS generation [21, 36, 37].

### Virus construction and infection

MicroRNA (miRNA) sequence miRNA-HO-1-166 (5′-TCCTCATGAACTCAGCATTCT-3′) targeting human HO-1 was selected with Invitrogen Block-iT RNAi designer software (www.invitrogen.com/rnai). pcDNA™6.2-GW/EmGFP-miR-neg plasmid from Invitrogen was used as a source for negative control miRNA expression. miRNA-encoding viruses were produced with Block-iT Lentiviral Pol II miRNAi expression system (Invitrogen) in 293FT cells. Lentiviral stocks were concentrated using Lenti-X™ concentrator and titers were determined with Lenti-X™ qRT-PCR titration kit (both purchased from Clontech, Saint-Germain-en-Laye, France). For infection, cells were plated onto 12 well plates at 2.5 × 10^5^ cells/well and infected with lentiviral stocks at an MOI of 10 in the presence of polybrene, then analysed by flow cytometry, RT-PCR and Western blotting 48 h post infection. Stably transduced cells were selected using Blasticidin selection marker (8 μg/ml) for 2 weeks before use.

## STATISTICAL ANALYSES

Student's T-test was used to assess the statistical significance compared to that of the control values. Results with a P < 0.05 were considered statistically significant and are indicated (*). Values represent the means ± SEMs from at least three independent experiments, or of representative blots/plots that are typical of at least three independent findings.
